# 

**Published:** 1906-11

**Authors:** 


VOL. XXII. NOVEMBER, 1906. No. 5.

NOV. 10 1906

### TEXAS MEDICAL JOURNAL

"RED BACK"

PUBLISHED AT AUSTIN, TEXAS, BY

F. E. DANIEL, M. D., - - Proprietor

PRINTED BY THE VON BOECKMANN-JONES CO.

**Subscription, $1.00 a Year, in Advance. - - - Single Copies, 10 Cents**

Entered at the Postoffice at Austin, Texas, as Second-class Mail Matter

